# Complete Cranial Iliac Osteotomy to Approach the Lumbosacral Foramen

**DOI:** 10.3389/fvets.2017.00075

**Published:** 2017-05-19

**Authors:** Barbara Dyall, Hugo Schmökel

**Affiliations:** ^1^Ryggcenter, Specialistdjursjukhuset Strömsholm, Strömsholm, Sweden

**Keywords:** canine, lumbosacral degeneration, foramen, surgical approach, osteotomy

## Abstract

An approach using a complete cranial iliac osteotomy (CCIO) to access the lumbosacral (LS) foramen in dogs from lateral was developed using cadavers and applied in a clinical patient with degenerative lumbosacral stenosis (DLSS). The foraminal enlargement in the cadavers and the patient was documented on postoperative CT scans. The preoperative CT scan of the patient showed moderate cranial telescoping of the sacral roof and a moderate central disk protrusion, leading to moderate to severe compression of the cauda equina. In addition, there was lateral spondylosis with consequential stenosis of the right LS foramen. The right L7 nerve had lost its fat attenuation and appeared thickened. After a routine L7S1 dorsal laminectomy with a partial discectomy, a CCIO was performed, providing good access to the LS foramen and the adhesions around the proximal L7 nerve caudoventral to the foramen. The osteotomy was stabilized with a locking plate and a cerclage wire. The dog recovered well from the procedures and after 36 h, the dog walked normally and was discharged from the hospital. Eight and 16 weeks later, the signs of the DLSS had markedly improved. From these data, it can be concluded that the CCIO is a useful approach to the LS foramen and intervertebral disk in selected patients with DLSS, giving good access to the structures around the LS foramen.

## Introduction

Degenerative lumbosacral stenosis (DLSS) is an important cause of chronic pelvic limb and spinal pain in dogs (1). In this condition, numerous soft and bony tissue alterations of the spine can be found, leading to often exercise-dependent compression of the cauda equina, including the nerve roots of the L6, L7, and S1 nerves in the spinal canal and/or foramina (2, 3). Most dogs are affected by intervertebral disk protrusion and proliferation of the interarcuate ligament dorsally, responding well to dorsal decompression and a partial discectomy. However, a considerable percentage of these patients also suffer from nerve compression within the neuroforamen, or nerve compression caused by tissue proliferation lateral to the neuroforamen (1, 3–6). It was the aim of this case report to investigate the feasibility and benefit using a complete cranial iliac osteotomy (CCIO) as a surgical approach to the lateral lumbosacral (LS) foramen in selected patients with DLSS.

## Materials and Methods

Five fresh canine cadavers were used for the preliminary study. All dogs were euthanized for other reasons than the study and used with owner’s consent. A CT scan was performed (Phillips Brilliance CT 40 Channel; Philips Healthcare Sverige, Stockholm, Sweden) in two cadavers using the cervical spine helical program, with 1-mm slices and 0.5-mm increments before and after the surgical procedure. Digital photographs were taken to document the surgical access to the LS foramen and nerve.

### CCIO Surgical Procedure

**Figure 1:**
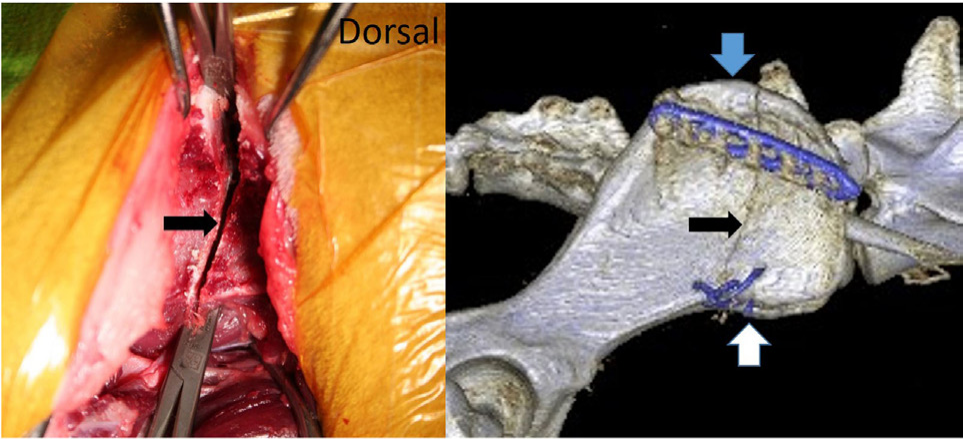
**Picture from a cadaver surgery and the postoperative CT scan of our patient showing the bidirectional osteotomy of the cranial iliac wing (black arrow)**. The medial gluteal muscle was prepared caudally, and blunt instruments were used to protect the soft tissues medial to the iliac wing during the osteotomy. The blue arrow marks the cranial dorsal iliac spine and the white arrow the ventral alar spine.

**Figure 2:**
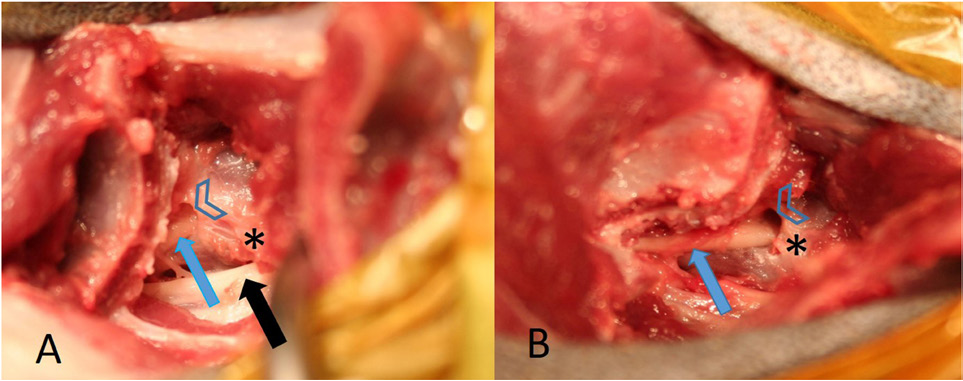
**(A)** from a healthy cadaver is showing access to the lumbosacral (LS) foramen after the complete cranial ilium was rotated cranially. The L6 nerve (black arrow) and the L7 nerve (blue arrow) can be identified. The arrow heads mark the cranial rim of the LS foramen and the star the caudal rim of the L7 transverse process. **(B)** is showing the contralateral L7 nerve followed more caudally by removing a small part of the ilium wing with a Kerrison punch forceps, the foramen was enlarged cranially.

The skin incision is placed over the cranial ilium. Starting from the cranial iliac rim, the medial gluteal muscle is prepared and elevated caudally from the ilium until the osteotomy line is reached. The attachment of the sartorius muscle can be left in place. The cranial dorsal iliac spine and ventral alar spine are the landmarks for the osteotomy (Figure 1). The dorsal and ventral fascia of the cranial dorsal iliac spine and ventral alar spine are incised, allowing the passage of a blunt instrument underneath the wing of the ilium (Figure 1). The place and extent of the osteotomy can be measured on the preoperative CT scan, and these measurements can be applied in surgery. To verify the correct osteotomy line, the cranial end of the sacral wing is identified by palpation with a blunt instrument medial of the ilium wing. A bidirectional osteotomy with a straight oscillating saw blade is performed using blunt instruments to protect the tissues underneath the iliac wing. The osteotomy follows the cranial outline of the sacral wing. The ventral part of the osteotomy should aim more caudal to allow good access to the L6 and L7 nerve (Figures 1 and 2).

**Figure 3:**
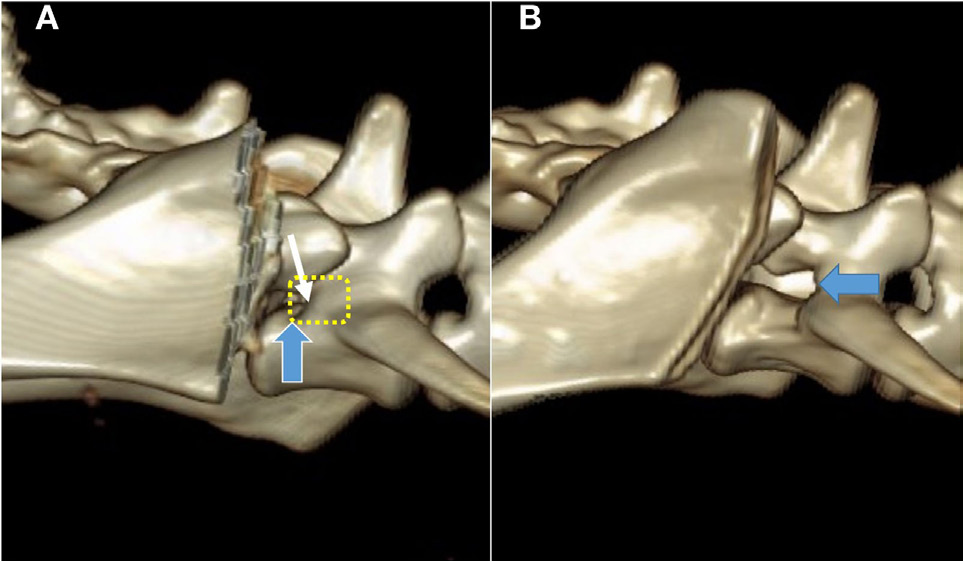
**Pre- (A) and post-procedure (B) 3D CT reconstructions of the stenotic lumbosacral foramen (blue arrow) of the cadaver described also in Figure 4**. The planed foraminal enlargement is shown by the yellow dotted line, and the cranial rim of the foramen by the white arrow.

The cranial part of the iliac osteotomy includes the insertions of the iliocostalis, the longissimus, and quadratus lumborum muscles. This part of the iliac osteotomy is reflected cranially to expose the L7 transverse process. The L6 nerve with the obturator branch is visualized running under the L7 transverse process (Figure 2). The dorsal base of the L7 transverse process is identified and visualized using a Freer periosteal elevator. After that the cranial rim of the LS foramen can be palpated and prepared until a Cushing nerve probe can be inserted gently behind the pedicle wall. The ventral pedicle bone between the cranial foraminal rim and the caudal base of the L7 transverse process is thinned using a high-speed drill with a 2–3 mm round burr (Figures 2 and 3). Then, the LS foramen is enlarged cranially using 1 and 2 mm Kerrison punch forceps. If necessary, a bigger foraminal enlargement can be planned to extend the opening more cranially and dorsally. The 3-dimensional CT reconstructions can be used to establish and measure the necessary enlargement to decompress the L7 nerve root. The L6 and L7 nerves are released from existing adhesions and can be covered with autologous fat. The osteotomy is realigned and stabilized with a locking plate, and a ventral cerclage wire can be placed additionally (Figure 1).

## Results

**Figure 4:**
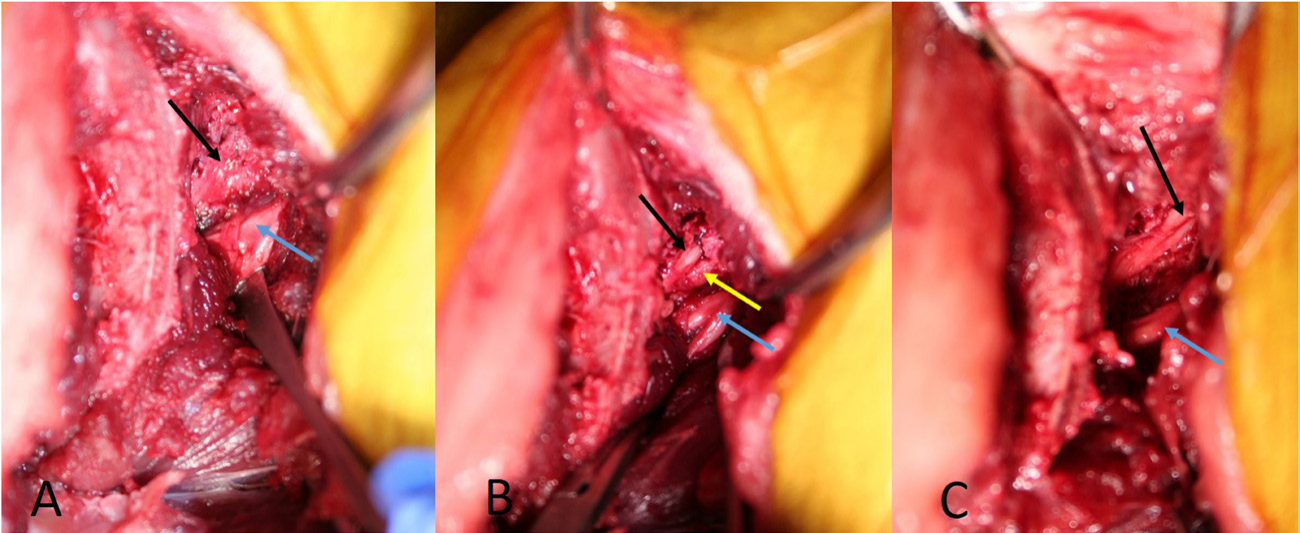
**Picture from the cadaver with foraminal stenosis**. **(A)** is showing the L7 nerve (black arrow) covered with fibrotic tissue. **(B)** is showing the L7 nerve (black arrow) leaving the stenotic foramen after dissecting the fibrotic tissue. **(C)** is showing the L7 nerve (black arrow) after enlarging the stenotic foramen. The blue arrows mark the L6 nerve, the yellow arrow marks the ventral rim of the stenotic foramen.

In all cadavers, the approach did not macroscopically damage the nervous tissues medially of the cranial ilium wing. After the transverse process of the L7 vertebra was identified and visualized, the cranial rim of the LS foramen could be palpated with a Cushing nerve probe (Figure 2). One cadaver had foraminal pathology with fibrous tissue covering the L7 nerve. Magnification was necessary in this case to carefully dissect the fibrous tissue and expose the foramen. The L6 and L7 nerve could be identified and protected with a probe, and the foramen was enlarged with the high-speed drill and Kerrison punch forceps in all cadavers, without macroscopically damaging the nerves (Figure 4). The control CT scan showed marked foraminal enlargement compared to the preoperative CT scan (Figure 3).

## Clinical Case

An 8-year-old spayed female German Shepherd was referred for investigation and treatment of lower back pain combined with lameness of the right pelvic limb. The joints of both pelvic limbs were normal, and mild lameness of the right pelvic limb together with mild muscle atrophy was detected. The neurological examination was unremarkable, but pain could be found during the palpation of the LS area. The radiographs of the referring veterinarian showed signs of DLSS.

**Figure 5:**
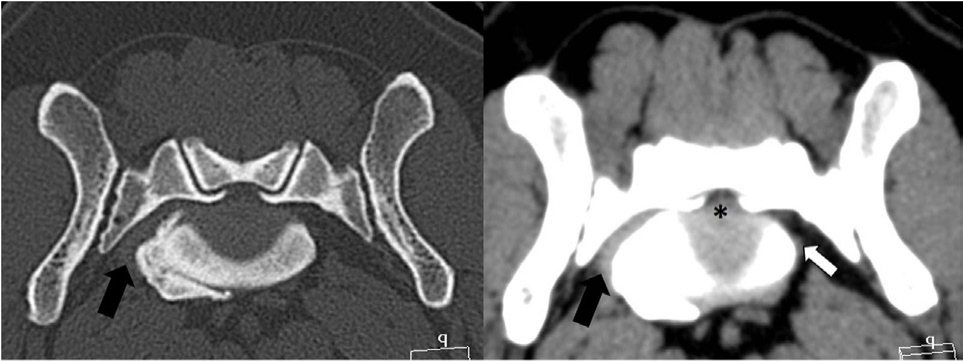
Transverse CT images of the bone and soft tissue window from the patient’s lumbosacral foramen showing a moderate disk protrusion (*) and a ventrolateral spondylosis, resulting in stenosis of the right foramen. The left L7 nerve is of normal size (white arrow); however, the right L7 nerve cannot be clearly identified within the soft tissue proliferation (black arrow).

The CT scan showed moderate cranial telescoping of the sacral roof and moderate central disk protrusion, leading to moderate to severe compression of the cauda equina. In addition, there was lateral spondylosis with consequential stenosis of the right LS foramen. The right L7 nerve had lost its fat attenuation and appeared to be thickened (Figure 5).

The patient was medically managed with activity restriction, gabapentin (10 mg/kg TID), and carprofen (2 mg/kg BID). Two weeks later, the dog was presented again because her condition had deteriorated, and she exhibited obvious difficulties in getting up and sitting down. A surgical treatment with a L7S1 traction-fusion procedure was discussed. But because such a stabilization does not allow visualization of the pathologies latero-ventral of the LS foramen, and the amount of the enlargement of the foramen by the traction is more unpredictable because of the tissue proliferations, the owner agreed to perform a dorsal L7S1 decompression, followed immediately by a right-sided CCIO.

### Surgical Procedure

**Figure 6:**
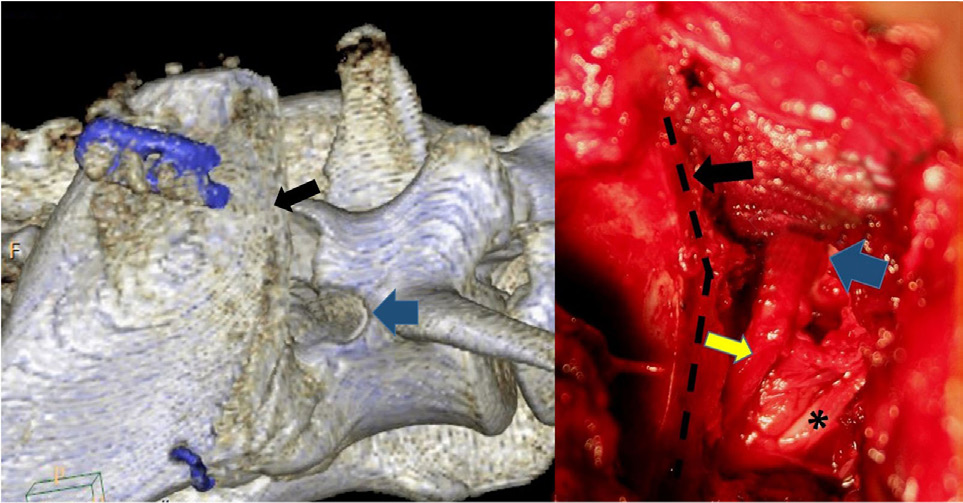
**The osteotomy (black arrow and black dotted line) has been repeated digitally on the postoperative CT scan to mimic the patient’s intraoperative procedure**. The blue arrows mark the cranial rim of the enlarged lumbosacral foramen, while the L6 nerve exhibits a normal structure (*). The L7 nerve (yellow arrow) has been freed of adhesions. The visible part of the nerve is increased in thickness.

The dog was premedicated with methadone (0.3 mg/kg subcutaneously) and acepromazine (0.05 mg/kg subcutaneously). After induction with propofol (4 mg/kg intravenously), carprofen (4 mg/kg subcutaneously) was administered, and anesthesia was maintained with isoflurane/O_2_. Standard equipment was used to monitor the anesthesia, and no antibiotics were given to this patient. During the L7S1 dorsal laminectomy, the cranial sacral lamina was removed to eliminate the dorsal nerve root impingement, and a partial annulectomy reduced the ventral cauda equina compression. After completion of the laminectomy, the patient was placed in left lateral recumbency, and the affected right pelvis was surgically prepared from the L4 to the greater trochanter. The CCIO procedure was performed as described earlier. The LS foramen was enlarged cranially. Fibrotic tissue covered the L7 nerve adhering it to the bone, making dissection with magnification lenses and micro-instruments necessary to mobilize the nerve from the sacral bone proliferations (Figure 6). The nerves were covered with autologous fat, and the osteotomy was realigned and stabilized with a dorsally placed 2.4 LCP locking plate, using the thicker dorsal ilium rim for screw anchoring, and a ventrally 1 mm cerclage wire to counteract the forces generated by the medially inserting muscles (Figure 5). A more ventrally placed locking plate may make the cerclage unnecessary. Then, the medial gluteal muscle was sutured back with single stitches using 3-0 absorbable monofilament, and a routine wound closure was performed using 3-0 monofilament suture material. The postoperative CT scan showed adequate dorsal decompression and enlargement of the right LS foramen.

### Postoperative Management

Intravenous Ringer lactate fluids at a rate of 4 ml/kg/h were continued after the anesthesia, together with a fentanyl constant rate infusion at a rate of 5 μg/kg/h. Carprofen (2 mg/kg BID) and gabapentin (10 mg/kg TID) were added the following day. The patient recovered quickly and could walk and urinate outside after 12 h. Thirty-six hours after the surgery, no opioids were necessary, the dog walked normally and was discharged with carprofen (2 mg/kg BID) and gabapentin (10 mg TID) for 4 weeks. Controlled rehabilitation using passive range movements, muscle massage, and a water treadmill was done twice a week. Four and eight weeks after the surgery, the dog was examined and showed no signs of pain, and the lameness had resolved. No complications have been noted from the iliac osteotomy. Radiographs of the pelvis at 8 weeks showed healing of the ilial osteotomy. Four months after the procedure, the dog was doing well, and no complications of the osteotomy were recorded by the referring veterinarian, the physiotherapist, or the owner.

## Discussion

When a patient presents with a suspected LS stenosis, a thorough clinical examination and work-up are important for localizing the lesion precisely within the LS area and to exclude any potential orthopedic causes. Although not specific, exercise-related pain and the exacerbation of lameness with the extension of the affected limb and LS spine are the clinical hallmarks of these patients. Palpation of the L7S1 area causes a pain reaction in most of the patients with DLSS. Neurological deficits like prolonged proprioception response or fecal incontinence can be found in advanced cases (3, 6, 7). Determination of spinal nerve impingement by imaging through CT or MRI is necessary for surgical decision making and for the selection of the surgical decompression techniques in dogs with DLSS and foraminal stenosis (8). One diagnostic difficulty in these cases is to differentiate between L7 and S1 nerve root lesions, because both supply the same myotomes of the sciatic nerve, with considerable individual variation among dogs (7, 9). Additionally, pathological changes, including foraminal bone proliferation, can be found in canine CT scans without corresponding clinical signs (10). Despite recent improvements in advanced imaging techniques, there remain difficulties in diagnosing the impingement of the L7 spinal nerve due to foraminal stenosis (8). The decision to perform a CCIO procedure must be based on the patient’s history, clinical presentation, and advanced imaging.

Different surgical approaches have been developed to alleviate L7 foraminal nerve compression. A dorsal foraminotomy with a facetectomy is not recommended because of the risk of increasing LS instability (7). A foraminotomy using endoscopically assisted instrumentation through a dorsal laminectomy can enlarge the inner part of the foramen, but the exit zone and the extraforaminal part of the L6 and L7 nerves cannot be addressed. A L7S1 traction-fusion procedure does not address extraforaminal pathologies (1, 4, 11) Moreover, the dorsolateral approach described by Gödde and Steffen allows the enlargement of the craniodorsal part of the L7 foramen, but the access is limited by the iliac wing (7). A partial, transverse iliac osteotomy removing the dorsal part of the ilium wing to increase the access has been described (12). The disadvantage of this osteotomy are the insertions of the iliocostalis and longissimus lumborum muscles left in place in the ventral part of the ilium wing, reducing the access to the L7 nerve and intervertebral disk (12). Another described approach can expose the L7 nerve and, after additional resection of the ventral aspect of the sacral wing, the L7S1 intervertebral disk (1). Because of the small size of the transiliac window and the deep position of the structures to be treated, direct visualization is difficult. An endoscopic technique allows improved observation, but for cases with large lateral tissue proliferation, it may not provide adequate access (1, 13).

A surgical approach using a CCIO is possible to perform, and the muscles inserting medial to the cranial ilium are reflected cranially with the osteotomized bone. No additional resection of these muscles is necessary, limiting the trauma to the non-weight bearing iliac wing. If it is necessary to follow the L7 nerve more caudally, a small part of the sacral wing can be excised to visualize and remove the bony proliferations often irritating the nervous tissue. Only the cranial aspect of the sacral wing, under the base of the articular process, needs to be excised. This part of the sacral wing does not contribute directly to the sacroiliac joint (14).

The patient treated with a dorsal laminectomy and a CCIO recovered fast, walking the next day showing no side effects from the procedures. Eight and 16 weeks later, the signs of the DLSS had markedly improved. In cases with dorsal and lateral procedures to decompress LS nerves, the effect of each single approach cannot be quantified, only the combined benefit for the patient. A staged treatment with a dorsal decompression first can be discussed with an owner, followed by a CCIO 6–12 weeks later if the signs of nerve impingement do not improve.

## Concluding Remarks

After preparing for this CCIO procedure using canine cadavers and the successful surgery performed in this first patient, we can conclude that the CCIO is a useful approach to the LS foramen and intervertebral disk, giving good access to the structures around the LS foramen.

## Ethics Statement

The cadavers used in this study were euthanized for reasons not related to the study, and the bodies were used with owner’s consent. The patient in this study was treated with owner consent. The owner was informed that the surgical procedure has not been done previously at our clinic.

## Author Contributions

BD contributed to writing the manuscript and the literature review. HS contributed to writing the manuscript, and he was the primary surgeon of the case.

## Conflict of Interest Statement

The authors declare that the research was conducted in the absence of any commercial or financial relationships that could be construed as a potential conflict of interest.
